# Age- and sex-associated differences in immune cell populations

**DOI:** 10.1016/j.isci.2025.113092

**Published:** 2025-07-10

**Authors:** Reza Gheitasi, Sabine Baumgart, Daniela Roell, Norman Rose, Carsten Watzl, Diana Dudziak, Nico Andreas, Oliwia Makarewicz, Sebastian Drube, Clara Schnizer, Thomas Kamradt, Sebastian Weis, Mathias W. Pletz, for the CoNAN study group

**Affiliations:** 1 Institute for Infectious Diseases and Infection Control, Jena University Hospital, Friedrich-Schiller-University Jena, 07747 Jena, Germany; 2 Department of Rheumatology and Clinical Immunology, Clinic of Internal Medicine III, University Hospital Bonn, 53127 Bonn, Germany; 3 Institute of Immunology, Core Facility Cytometry, Jena University Hospital, Friedrich-Schiller-University, 07743 Jena, Germany; 4 Institute of Immunology, Jena University Hospital, Friedrich-Schiller-University, 07743 Jena, Germany; 5 Department of Immunology, Leibniz Research Centre for Working Environment and Human Factors at the Technical University Dortmund (IfADo), 44139 Dortmund, Germany; 6 Leibniz Institute for Natural Product Research and Infection Biology, Hans-Knöll Institute (HKI), 07745 Jena, Germany; 7 Department of Anesthesiology and Intensive Care Medicine, Jena University Hospital, Friedrich-Schiller-University, 07743 Jena, Germany

**Keywords:** Components of the immune system, Immunology

## Abstract

Aging is associated with the risk of increased infection severity and altered immune responses. In this study we investigated age- and sex-specific differences in immune cell composition in a subset of the population-based CoNAN study using a cross-sectional analysis. We identified a significant age × sex interaction in memory B cells and observed age-related declines in naive lymphocytes and an increase in CD8^+^ effector memory T cells in men. Additionally, numbers of dendritic cell subpopulations decreased with age in both sexes. This study provides new insights into complex dependencies of the immune cell composition on age and sex (e.g., age × sex interaction effects) and could enhance our understanding of immune status variations among different ages. However, further studies are needed to assess the functional implications of these compositional differences.

## Highlights

• Age × sex interaction in immune cell composition revealed by mass cytometry
• Granulocyte composition consistent across sexes and ages
• Memory B cell increase in elder women drove age × sex interaction
• CD8^+^ T cell subsets show sex-specific age-related changes

## Graphical abstract

**Figure:**
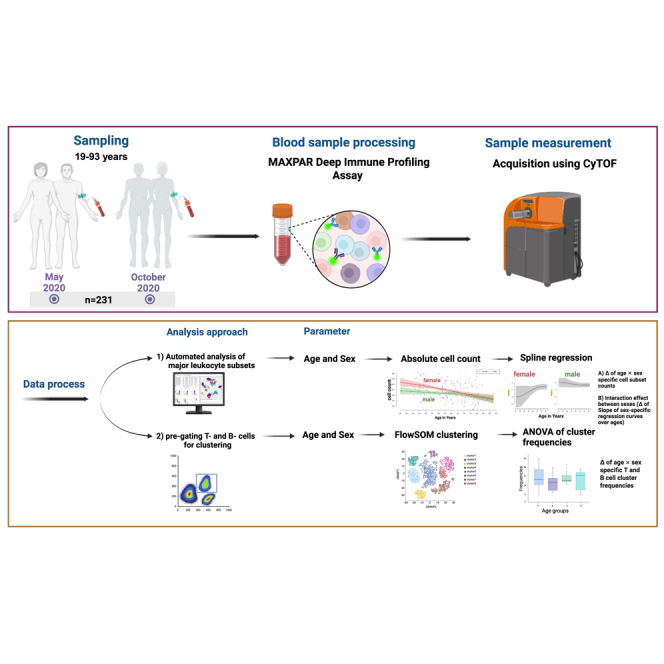

## Introduction

The aging process primes gradual dysregulation of immune functions, affecting most components of the immune system and heightens the susceptibility to infections, autoimmunity, and impacting vaccine efficacy due to waning immune responses.^1^^,^^2^ Due to the intricate relationships between overall health, sex, and aging,^3^ it is valuable to understand how age and sex, respectively, influence the immune system. Studies have reported distinct alterations in several types of immune cells according to the age of the individual,^4^ but (1) do not stratify by sex, despite sex-specific differences in immune cell composition are well known and (2) often assign study subjects to pre-defined age groups, which may result in missing immunosenescent dynamics within the predefined age-groups.^5^^,^^6^ Also, sex -specific differences have been observed, with females showing higher numbers of total leukocytes, including higher proportions of T cells, B cells, and macrophages in tissues like the peritoneal and pleural cavities compared to males.^7^ Other studies have addressed sex-specific differences but omitted age as modifying factor, or interaction of age- and sex-on changes within leukocyte subpopulations.^8^^,^^9^^,^^10^ However, with human lifespan extension the risk of diseases with enhanced severity increases.^11^^,^^12^ It has been shown that aging is a major risk factor of susceptibility to chronic obstructive pulmonary disease (COPD), fatal respiratory infection, and primary lung cancer.^13^^,^^14^^,^^15^ The aging effect on immunity encompasses a variation in the function and distribution of immune cells involved in both, innate and adaptive immunity.^16^ Aging-related research focused on the adaptive immune system, especially T cells which have been studied more than other adaptive immune cell subsets.^17^^,^^18^ Thymic involution as a major change related to age in the immune system has been shown to cause variations in the number of naive subsets of T cells.^19^ In this study, we investigated the age- and sex-specific development in different immune cell lineages over age without pre-specified grouping by using high dimensional cytometry on samples of the population-based prospective cohort study (CoNAN, German Clinical Trials Register: DRKS00022416),^20^ to address: (1) overall age-related changes of immune cell phenotype composition regardless of sex, (2) age-related differences and comparisons of immune cell phenotype composition between sexes, and (3) age × sex interaction influence on immune cell phenotype composition.

## Results

### Study population and single-cell flow cytometry analysis

**Figure 1:**
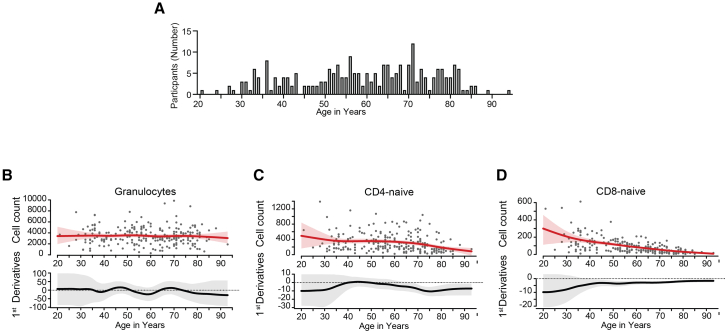
Age distribution and spline regression analysis of granulocyte and naive T cell counts across the human lifespan (A) Age distribution of participants (*n* = 236). Absolute counts of (B) granulocytes, (C) CD4^+^ naive T cells, and (D) CD8^+^ naive T cells of all intact leukocytes. Each dot indicates an individual participant. Red lines with the 95% confident band indicate spline regression, black lines with the 95% confident band indicate the first derivative of the spline regression. (Supplementary information on graphs interpretation exemplary on (D): Flexible spline regression model was used to analyze cell count trends across the lifespan, avoiding the need for pre-defined age groups or assumptions about the relationship between cell numbers and age. The upper panel depicts the overall negative trend in naive CD8^+^ T cell counts with increasing age. The lower panel shows the age-specific slopes of the regression curve, along with their 95% confidence intervals, illustrating the rate of change in cell counts at different ages. A slope significantly different from zero, where the confidence interval does not overlap with zero, indicates a statistically significant increase or decrease at that specific age. In this case, a significant decline in naive CD8^+^ T cell number is evident from age 38 onwards. Notably, the varying slope across the age spectrum reveals subtle fluctuations in the rate of decline over time).

This study investigated the impact of sex and age on innate and adaptive immune cells of 231 participants, 117 women (time point 1 = 59, time point 2 = 58) and 114 men (time point 1 = 58, time point 2 = 56) between the age of 19 and 93 years (Table S1) without acute or chronic infection. Participants filled out a pseudonymized questionnaire on-site during all three study rounds. Demographic data underwent plausibility checks to ensure accuracy, and are presented in Table S1 (BMI values are presented in Figure S5). Peripheral blood was taken at two time points, in May or October 2020, respectively. Only subjects with seronegative and negative PCR test against SARS-CoV-2 (<4 out of six different tests) at the earlier time point (May 2020), and subjects with no symptoms for respiratory infects at the later time point (October 2020) were enrolled in this study. Considered exclusion criteria were based on CoNAN cohort,^21^ and all acutely ill subjects were not enrolled in this study. By combining mass cytometry that allows deep phenotyping of immune cells to the level of certain subpopulations and a unique statistical method, we identified several age- and sex-specific differences in the composition of these subpopulations over a variety of ages (Figure 1A).

We analyzed whole-blood samples of male and female^22^ participants and obtained absolute counts of major CD45^+^ leukocyte subsets phenotype^23^ (For further phenotype characteristics see, Table S2). The overarching question of our study concerns the aging of the immune system over the lifespan. Accordingly, we use the terms change, increase, and decrease throughout the manuscript when describing and interpreting the results. Additional assumptions are required to interpret the relationship between age and the cross-sectional cytometric data as change. The key assumption is that the stochastic relationship between age and cell counts is invariant across all birth cohorts, both in the total population and within the two sex groups. This is an untestable assumption in our sample, which needs to be kept in mind when interpreting the results.

### Age-related changes in immune cell phenotype composition

To determine differences in cell-type composition in different ages, we first compared the absolute numbers of each cell population from young (19 years) to aged adults (93 years) (Figure 1A) regardless of sex, using age as a continuous variable. We did not find relevant differences in absolute numbers of CD66b^+^ granulocytes (Figure 1B), and total monocytes (predominantly classical monocytes) across different ages (Table S3).

#### Innate immune cell age-related differences

Within monocyte subsets significant increases in the number of non-classical monocytes were seen across the age range of 20–57 years. Transitional monocytes showed a statistical significant increase specifically within the narrow age range of 63–64 years (Table S3). Other innate immune cells such as natural killer cells (early and late NK) showed a statistical significant increase during two age ranges, between 30–33 and 63–66 (Table S3). Among dendritic cells (DCs), plasmacytoid (pDCs), and conventional DCs (cDC) numbers were both decreased in late age (pDC: age 75–93; cDC: age 81–93) (Table S3).

#### T-lymphocytes age-related differences

The adaptive immune system cells exhibited distinct alterations, particularly evident in T cell composition, which showed statistically significant variability across different age ranges of individuals involved in the study. CD4^+^ and CD8^+^ T cells decreased at middle age almost at the same lifetime (CD4^+^: age 58–63; CD8^+^: age 53–55). A second statistical decrease in CD4^+^ T cells was observed between the ages 69 and 75. Among naive T cell subsets, CD8^+^ naive T cells significantly decreased much earlier in life, starting from the age of 38 until 93 (Figure 1D), compared to the decrease in CD4^+^ naive T cells which occurred later in life (age 69 to 93) (Figure 1C). In contrast, there was a notable significant decrease in CD4^+^ and CD8^+^ effector memory T cells (T_em_) counts between the ages of 56–75 and 53–55, respectively. Conversely, the terminally differentiated effector memory RA^+^ (TEMRA^+^) subset of CD4^+^ T cells showed alternating statistical significant increases at ages 20–35, 43–49, and 68–72, while the subset of TEMRA^+^ CD8^+^ T cells increased only in earlier and later age spans (ages 20–36 and 61–74) (Table S3). Our findings show a statistical significant reduction in major CD4^+^ helper T cell subsets, including Th1 and Th17 cells, occurring between the ages of 55–60 and 57–72, respectively. Conversely, CD4^+^ Th2 cells showed a statistical significant increase during middle age, between 46 and 53 years (Table S3). The CD4/CD8 T cell ratio showed a statistically significant age dependent pattern, peaking during middle age (46–56 years) and subsequently declining in older adulthood (66–93 years) (Table S3). Additionally, other T cell subsets such as mucosa-associated invariant T cells/natural killer T cells (MAIT/NKT) significantly decreased over the age range of 37–52, while γδ T cells showed an initial increase between ages 40–49, followed by a statistical significant decrease in the elderly period (82–93 years) (Table S3).

#### B- lymphocytes age-related differences

Analysis of B cell subpopulations depicted a statistical significant decrease in the number of plasmablasts above the age of 67, persisting into late life age range (up to 93 years old) (Table S3).

Overall, these results suggest that along with dwindling DC subsets and plasmablasts, the T cell profile shifts from naive into the effector memory stage with increased age of the participants.

### Age-related changes in immune cell phenotype composition within sexes

To determine immune cell differences within sexes in different ages, we analyzed cell subset numbers using smoothing spline regression.

#### Age-related changes in immune cell phenotypes in females

Non-classical monocytes in women showed a statistically significant increase from young to middle age, starting from 20 to 61 years (Table S4). Late NK cell numbers significantly increased early in life in female participants, starting at 20 years until 72 years. A reduction in pDC counts was also observed among female individuals within 75–93 years (Table S4).

Analysis of CD3^+^ T cells indicated sex-specific differences, with women showing a significant reduction between age 47 to 72 years, driven by significantly decreased CD8^+^ T cells (ages 20–53) and CD4^+^ T cells (ages 54–93), respectively. The Th2 phenotype of CD4^+^ T cells showed statistically significant increases from ages 20 to 49. Analysis of the C4/CD8 ratio showed a statistical significant increase in females between the ages of 20–52 (Table S4). Unconventional T cells showed a significant decrease in γδ -T cells between ages 33–41, as well as a significant reduction in MAIT/NKT numbers spanning from 20 toward 93 years of age in women.

Lastly, assessing of sex-related compositional differences in B-lymphocytes showed a stronger statistical significant decrease in the number of memory B cells (B_mem_ cells) from age 20 to 80 years in women (Table S4).

#### Age-related changes in immune cell phenotypes in males

In men, non-classical monocyte numbers significantly increased with age, starting from individuals aged 20 to 57 years (Table S4). Additionally, the number of late NK cells significantly increased at later age of life in men, not before 59 years, and extended until 93 years of age. The observed significant decrease in total DC counts in men was primarily driven by lower counts of cDC between ages 65–93 (Table S4).

A significant reduction in CD4^+^ T cells was observed, between ages 59–93. Further analysis of T cell subsets revealed a statistical significant decrease in naive T cells in males, occurring between ages 52/55–93 for the CD4^+^ T cell subset and spanning from 20 to 93 years for CD8^+^ T cells (Table S4). The Th2 phenotype of CD4^+^ T cells showed significant increases during middle age (50–54). Furthermore, the amount of central memory CD8^+^ T cells (T_cm_) showed a sex-related significant increase solely in young male individuals, aged 20–46 years (Table S4). The CD4/CD8 T cells ratio in males showed a statistically significant reduction during older adulthood of 65 and 93 (Table S4).

#### Age-related immune cell changes comparing sexes

Next, we assessed the immune system status between the sexes. For this we calculated the mean difference of absolute cell numbers for each immune cell subset in peripheral blood of male and female participants.

We found no significant difference in the total number of granulocyte subpopulations i.e., neutrophil, basophil, or eosinophil granulocytes (data not shown).

**Figure 2:**
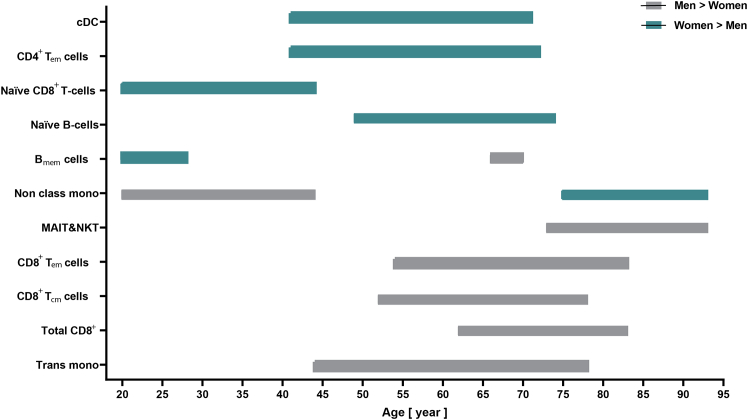
Comparison of absolute immune cell counts between sexes across the lifespan Each cell in the graph represents the mean difference in absolute count for a specific immune cell type (y axis) at a given age (x axis). Green indicates a significantly higher mean count in women compared to men, while gray indicates a significantly higher mean count in men compared to women. T_cm_: central memory, T_em_: effector memory, B_mem_ cells: memory B cells, MAIT/NKT: Mucosal associated invariant T cells/natural killer T cells, trans mono: transitional monocytes, cDC: conventional dendritic cells, non class mono: non classical monocyte, All values are indicating Year.

However, within innate immune cells, certain subsets showed distinct patterns between the sexes. Transitional monocytes (*Trans*-mono) and cDC showed significantly higher counts over a broad age range in men (*Trans*-mono: ages 44–78, cDC: ages 41–71). Non-classical monocyte counts peaked at age 20 in men. Later in life women showed significantly higher counts of non-classical monocyte population between the ages of 75 and 93 (Figure 2).

Comparing of T cell differences between sexes, we found a significant higher number of total CD8^+^ T cells in men aged between 62 and 83 years, alongside significant increasing counts of CD8^+^ T_mem_ cells in middle age, including CD8^+^ T_cm_ cells (aged 52–78) and CD8^+^ T_em_ cells (aged 54–83) (Figure 2). Interestingly, we observed significant higher counts of naive CD8^+^ T cells in young females (aged 20–44 years) (Figure 2), Within CD4^+^ T cells, CD4^+^ T_em_ cell count was significantly higher between the ages of 41–72 for female participants (Figure 2). Furthermore, among unconventional T cells, the number of MAIT/NKT statistically significant increases in men at late lifetime from 73 to 93 years compared to women. Across the study population, the CD4/CD8 T cell ratio remained significantly higher in females from middle age through older adulthood (51–93 years) (Table S5).

B cell subpopulations mean differences among male and female participants showed that naive B cells had a significantly higher count in middle aged women within the age range of 49–74 years. B_mem_ cells were more abundant in young women (aged 20–28 years), while in later life, men showed a significantly higher count of B_mem_ cells between 66 and 68; 70 years of age (Figure 2).

Our findings show that women experience differences of distinct immune cells already in early lifetime by elevated naive CD8^+^ T cell and B_mem_ cells counts while in men differences of immune cell counts were postponed to middle age until late-age. These differences were predominantly characterized by increasing T_mem_ cells, B_mem_ cells, MAIT/NKT, transitional monocyte, and cDC counts.

### Age × sex interaction effect on immune cell phenotype composition

**Figure 3:**
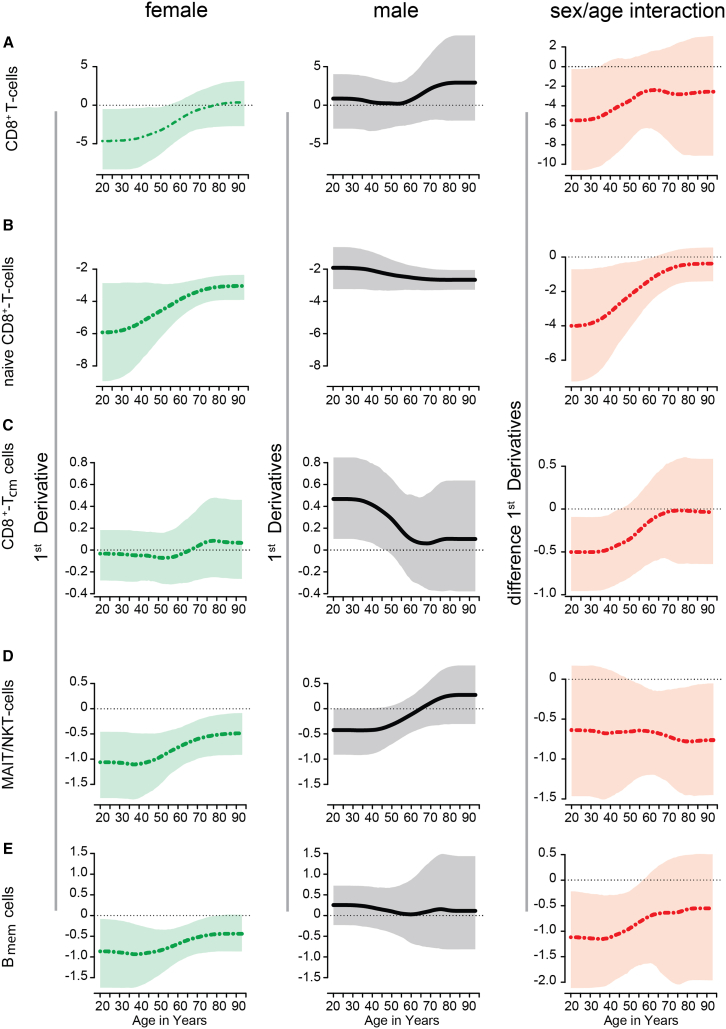
First derivatives of sex-specific spline regressions and age × sex interaction for selected immune cell populations First derivatives of sex-specific spline regressions of cell counts (left and middle column) and age × sex interaction effect (right column) representing the difference of the first derivative of the two sex-specific regression curves with 95%-confidence band, respectively, for (A) total CD8^+^ T cells, (B) naive CD8^+^ T cells, (C) CD8^+^ T_cm_ cells, (D) MAIT/NKT, and (E) B_mem_ cells in regard to age.

We compared males and females regarding the relationship between age and sex in immune cell counts to study possible interaction effects. Using non-parametric spline regressions, an interaction effect between age and sex with respect to cell counts is indicated by different slopes (e.g., first derivatives) of the sex-specific regression curves. In detail, we found an interaction effect between age and sex in the number of naive CD8^+^ T cells, CD8^+^ T_cm_ cells, MAIT/NKT, and B_mem_ cells (Figure 3), implying a stronger decline in the number of these immune cell populations in women compared to men at distinct ages (Figure S3).

#### Innate immune cells age × sex interaction

No age × sex interaction effect was found neither for granulocytes (i.e., neutrophil, basophil, and eosinophil), nor for other innate immune cell populations, such as monocytes or DCs.

#### T cells age × sex interaction

A statistical significant decrease of naive CD8^+^ T cells for both sexes over the examined lifetime (aged 20–93) was stronger among women compared to men between age 20 and 62. Taking into account the higher naive CD8^+^ T cell counts in younger women (20–44 years), and their prominent decline, the mean number of naive CD8^+^ T cells in both sexes converged at the age of 62 (Table S4). Additionally, CD8^+^ T_cm_ cell counts showed an age × sex interaction effect between 20 and 45 years. This effect is primarily driven by a slight but statistically significant increase in this T cell subtype among men aged 20 to 46, whereas no notable difference in mean CD8^+^ T_cm_ cell counts was observed among women in this age range. Accordingly, a higher level of this cell population in men between 52 and 78 years compared to women (Figure 3C; Table S4) is present. Alteration in CD8^+^ T cell subpopulations leads to a statistical significant decrease in the number of total CD8^+^ T cells only in women between 20 and 53 years. Hence, the interaction effect between sex and age regarding the number of total CD8^+^ T cells from ages 20 and 33 (Figure 3A; Table S4), indicating a stronger decline in total CD8^+^ T cell counts during early adulthood among women compared to men. Subsequent analyses of age × sex interaction uncovered a steady decrease pattern in MAIT/NKT over ages in female participants, spanning from age 20 to 93, with a notable acceleration toward late age (aged 50–93) (Figure 3D; Table S4).

#### B cells age × sex interaction

Furthermore, we found an age × sex interaction effect in the B_mem_ cell subpopulation between ages 20 to 56. This interaction effect is due to an average decline of B_mem_ cells among women between ages 20 to 80, whereas B_mem_ cell counts among men were age-independent (Figure 3E; Table S4).

### Deep immunophenotyping of T- and B-lymphocytes

**Figure 4:**
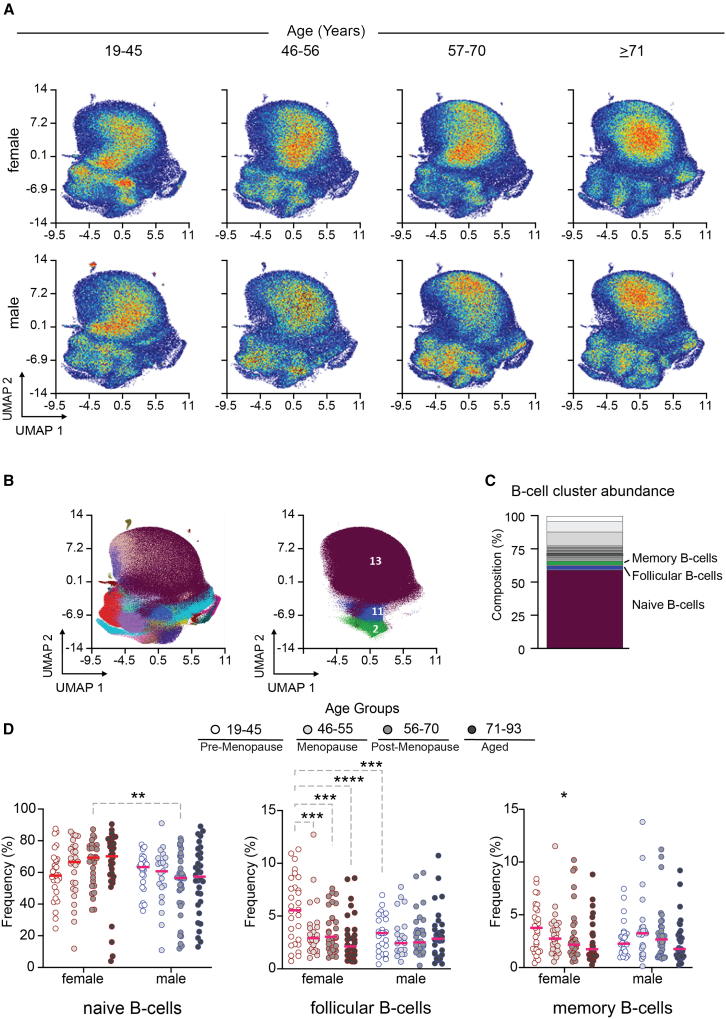
Differences in B cell composition across different sex and age groups (A) UMAP projection of pre-gated B cells from women and men in pre-defined age groups regarding the sexual hormone status of women in general. Each dot represents a single cell. Color represents the density of cells derived from age group 19–45 years (*n* = 29 females, *n* = 26 males), age group 46–56 years (*n* = 26 females, *n* = 21 males), age group 57–70 years (*n* = 30 females, *n* = 35 males), and age group 71–93 years (*n* = 32 females, *n* = 32 males). (B) UMAP projection of B cells. Color represents 20 different meta clusters derived from FlowSOM clustering (left) and selected clusters 2 (memory B cells), 11 (follicular B cells) and 13 (naive B cells) (right) that were statistically significant in age/sex interactions depicted in Figure 3D. (C) Bar chart shows the composition of all B cell clusters derived from FlowSOM clustering naming selected cluster 2, 11, and 13. (D) Graphs show frequencies of memory, follicular and naive B cells across different sex and age groups. Asterisks denote statistically significant results after applying ANOVA statistical test (∗*p* < 0.05, ∗∗*p* < 0.01, ∗∗∗*p* < 0.001, ∗∗∗∗*p* < 0.0001). Abbreviations: UMAP: uniform manifold approximation and projection, FlowSOM: self-organizing maps for cytometry data, ANOVA: analysis of variance.

**Figure 5:**
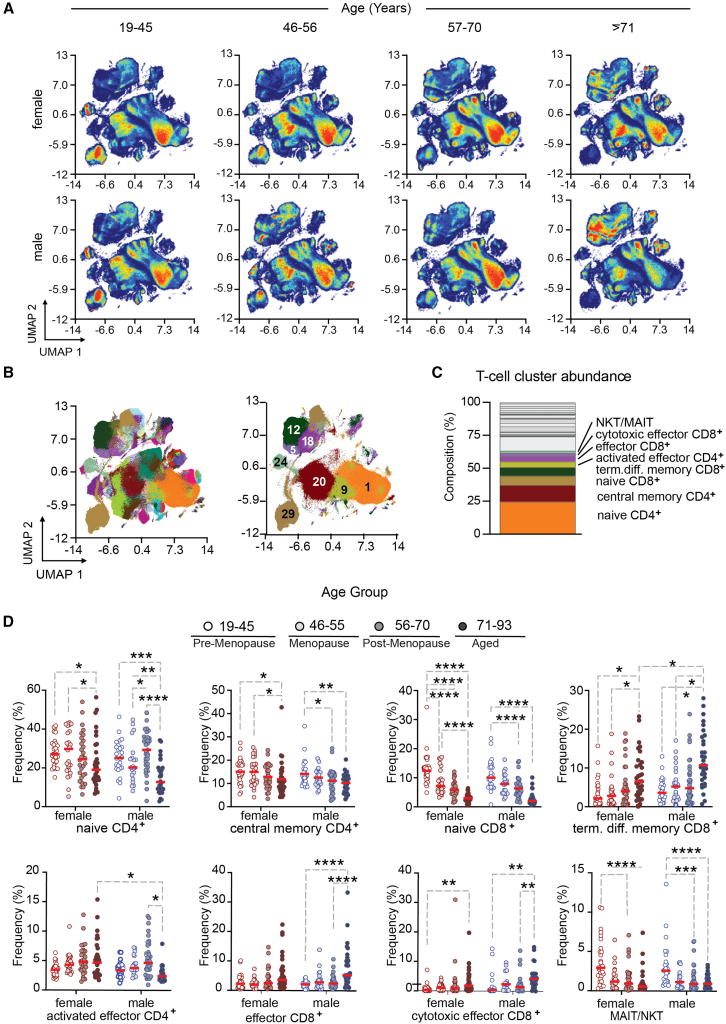
Differences in T cell composition across different sex and age groups (A) UMAP projection of pre-gated T cells from women and men in pre-defined age groups regarding the sexual hormone status of women in general. Each dot represents a single cell. Color represents the density of cells derived from different age groups consistent to Figure 3. (B) UMAP projection of T cells. Color represents 30 different meta clusters derived from FlowSOM clustering (left) and selected clusters 1 (naive CD4^+^ T cells), 5 (cytotoxic effector CD8^+^ T cells, 9 (activated effector CD4^+^ T cells), 12 (TEMRA CD8^+^ T cells), 18 (CD8^+^ T_eff_ cells), 20 (CD4^+^ T_cm_ cells), 24 (MAIT/NKT), 29 (naive CD8^+^ T cells) (right) that were statistically significant in age/sex interactions depicted in Figure 4D. (C) Bar chart shows the composition of all T cell clusters derived from FlowSOM clustering naming selected cluster from Figure 4B. (D) Graphs show frequencies of selected T cell clusters from Figure 4B across different sex and age groups. Asterisks denote statistically significant results after applying ANOVA statistical test (∗*p* < 0.05, ∗∗*p* < 0.01, ∗∗∗*p* < 0.001, ∗∗∗∗*p* < 0.0001). Abbreviations: UMAP: uniform manifold approximation and projection, FlowSOM: self-organizing maps for cytometry data, ANOVA: analysis of variance, Term.diff. memory: terminally differentiated memory, MAIT/NK: mucosal associated invariant T cell/natural killer T cell.

So far, we revealed distinct differences in the composition of major T- and B-lymphocyte populations during different ages in male and female participants. To investigate these cell populations phenotype characteristics in greater depth, we employed unsupervised self-organizing map (FlowSOM) clustering analysis and visualized the resulting cellular compositions using Uniform Manifold Approximation and Projection (UMAP) (For further phenotype characteristic see, Table S6). While ensuring a balanced distribution of the number of participants in each group for subsequent statistical analysis, we also considered already investigated hormonal effects on immune cell composition for the age range definitions.^7^ The defined age groups were, one pre-menopausal (aged 19–45 years), one associated to menopausal state in women (aged 46–56 years), one group post-menopausal (aged 57–70 years) and lastly aged adults (71–93 years) for further comparison^24^ (Figures 3A and 4A). Density plots of UMAPs for B cells in Figure 4A and T cells in Figure 5A showed differences across age ranges and sex.

#### In-depth immunophenotyping of B cells

Three out of the 20 obtained meta clusters, namely 2, 11, and 13 (Figure 4B) which are characterized by CD45, CD19, and CD20 co-expression, showed significant differences when performing statistical analysis on age groups using one-way-ANOVA (Figure 4C). In detail, the frequencies of B cell clusters 2 and 11, characterized by the expression of CD27^+^IgD^−^CD45RA^+^CXCR3^+^CXCR5^+^CCR6^+^HLADR^+^, showed a significant decrease only in women, albeit in different compositions. While cluster 2 (B_mem_ cells)^25^ declined not until the late age of 71–93 years, the proportion of cluster 11 dropped already two decades earlier, at the ages of 46–56 (Figure 4D). Cluster 11 (follicular B cells)^26^^,^^27^ differed phenotypically from cluster 2 by a higher expression of CD38 and CCR7 (Figure 4C). When considering sex-related differences, a significant higher frequency of cluster 11 was observed among women aged 19 to 45 compared to men. In addition, the frequency of cluster 13 (naive B cells)^28^ with the phenotype of CD27^−^IgD^+^CD45RA^+^CXCR3^−^CXCR5^+^CCR6^+^HLADR^+^CCR7^+^CD38^+^ was significantly higher in women than in men between age 56 and 70 (Figure 4D).

#### In-depth immunophenotyping of Tcells

Unbiased clustering analysis of T cells (Figure 5A) characterized by CD45 and CD3 revealed eight statistically relevant clusters (Figure 5B). Two T cell clusters 9 and 12 were detected with a statistically significant different sex interaction predominantly at late age above 71 (Figure 5C). While the proportion of cluster 9 assigned to activated CD4^+^ T_eff_ cells^29^ was higher in women in this age range, whereas the frequency of cluster 12 with a CD8^+^ TEMRA phenotype^30^ was lower in women compared to men (Figure 5D). When comparing naive CD4^+^ T cell cluster 1^27^ and activated effector CD4^+^ T cells in the group of men, we noticed a significant increase of naive CD4^+^ T cells in the age group of 56–70 but no change of activated effector CD4^+^ T phenotype, however, both clusters decreased with increasing age (Figure 5D). Further we observed that clusters 5 (cytotoxic effector T,^31^ and CD8^+^ TEMRA cells) and 18 (CD8^+^ T_eff_ cell^32^) showed a similar increasing tendency in both sexes. However, a reversed tendency was observed in CD4^+^ T_cm_ cells (cluster 20), MAIT/NKT (cluster 24) and naive CD8^+^ T cells (cluster 29) (Figure 5D).

A relevant question arises whether significant altered clusters identified through deep immunophenotyping exhibit similar patterns in the age × sex interaction analysis. To address this, we applied smooth spline regression to the identified clusters.

#### In-depth immunophenotyping of B cells age × sex interaction

We found that follicular B cells (cluster 11) displayed a higher frequency in females between ages 20–57 compared to males. However, in females, the frequency of follicular B cells showed a continuous decline from age 27 to 93, whereas no significant change was observed in males over lifetime (Figure S4A). While naive B cells (cluster 13) showed a lower frequency in males compared to females between ages 20–53, the decline in naive B cells in males between ages 20–56 contributed to the observed age × sex interaction (Figure S4B). Cluster 2 which was significantly altered, did not show any interaction between age and sex (Table S5).

#### In-depth immunophenotyping of T cells age × sex interaction

Among the significant identified T cell clusters from deep phenotyping, an age × sex interaction was observed only in activated effector CD4^+^ T cells (cluster 9) but no such interaction was detected in other clusters (cluster 1, 5, 12, 18, 20, 24, and 29) (Table S5). This population showed a marked increase in females between ages 20 to 65, whereas in males, a progressive decline was evident starting at age 65, continuing into later life (93 years). This sex-specific divergence was the primary driver of the observed age × sex interaction from ages 43 to 93 years (Figure S4C). It should be noted that females between 64 and 93 years old showed a significantly higher frequency of active effector CD4^+^ T cells.

## Discussion

Clinical studies often overlook the interplay between age- and sex-related immune differences, hindering our understanding of their combined impact on immune cell composition and maybe the outcome.^7^^,^^25^^,^^33^ The main observations of our study were age- and sex-impact on the composition of lymphocyte subpopulations, while granulocytes remained unaffected.

Previous studies addressing immuno-aging have reported on the composition and alteration in the proportion of specific immune cell subpopulations, such as T cells with age-grouped individuals.^4^^,^^26^ However, focusing solely on individual immune cell composition may not fully capture the complexity of the immune network. We here focused on a comprehensive approach by measuring concomitantly the absolute number of major circulating immune cell types in peripheral blood, not in pre-specified groups but in a continuous manner of a wide age range from 19 to 93 years. Our cohort was enrolled in 2020 during a COVID-19 field study (CoNAN study).^21^ CoNAN aimed to assess the seroprevalence in a village of approximately 900 inhabitants that had been quarantined after detection of the first COVID-19 case in this region. However, the analysis revealed that only a few of the enrolled subjects were seropositive, whereas 231 healthy individuals had been enrolled in this immunophenotyping study. At the time of enrollment none of the participants had been exposed to the pathogen or vaccinated against SARS-CoV-2, as vaccines were not yet available.

We here used mass cytometry to describe the dynamics on a more comprehensive and more detailed subpopulation level than conventional flow cytometry measurement. Unlike previous studies, we used statistical analysis to delineate sex-specific differences across various ages of participants without relying on pre-specified or artificial fixed age groups; instead, age was defined as a continuous variable.

Studies have shown that native cellular and humoral immune responses to antigen, vaccination, and infection are at a higher level at early age in women compared to men.^28^^,^^29^ It appears that men show an earlier immune-aging compared to women, characterized by earlier declines in naive lymphocyte populations and T cell proliferative capacity.^30^ In line with previous findings,^7^^,^^33^ our results confirm this sex-specific pattern. Despite age × sex interaction indicating a faster reduction in naive CD8^+^ T cells in women, we noted higher overall counts of naive phenotype compared to men, particularly in early to middle age. Using spline regression, we observed a consistent age-related decrease of either naive CD4^+^ or CD8^+^ T cell subsets. We did not detect any significant hormonal-associated differences in women. It can be argued that hormonal alterations in women may not exert a foremost independent effect on this specific T cell subset composition. Since naive phenotype represents an immature lymphocyte state that has not yet encountered antigens^27^ and women tend to have higher CD4/CD8 ratio than men,^31^ our findings imply that women could have a greater pool of potentially responsive naive T cells. An expanded capacity might offer advantages in mounting robust responses to new pathogens, potentially influencing disease susceptibility and vaccine efficacy.^32^ Hence potentially it might be beneficial to re-assess the current age-based recommendations for pneumococcal vaccines by World Health Organization (WHO) for individuals aged 60–65 years,^34^ exploring whether men might have an advantage from earlier vaccination compared to women.

Immune-aging of the adaptive immune system is often characterized by the expansion of antigen-specific effector and memory lymphocytes.^35^^,^^36^^,^^37^ We found significantly higher CD8^+^ T_cm_ cells in older men (52–78 years). In aging, with a reduction in naive T cells, T_cm_ cells become a more prominent subpopulation.^38^ T_cm_ cells are crucial for eliminating intracellular pathogens.^38^^,^^39^ The CD4/CD8 T cell ratio is a critical indicator of immune system health, reflecting the balance between helper and cytotoxic T cells. In females, we observed a statistically significant increase in the CD4/CD8 ratio (20–52 years), while males exhibited a significant decline (from 51 to 93 years). This finding aligns with existing literature suggesting that women sustain a higher CD4/CD8 ratio compared to men.^40^ This sustained higher ratio in women may contribute to their generally more robust immune responses; however, it could also predispose them to higher susceptibility to autoimmune conditions.^41^ Moreover, studies indicate that antigen-specific expansion of T_cm_ cells in elderly results in heightened production of interferon gamma (IFNγ) and tumor necrosis factor (TNF) compared to their adult counterparts, leading to improved survival rates in *Listeria monocytogenes* infection.^42^ Thus, higher CD8^+^ T_cm_ cells in men may bolster their cellular immunity against distorted or infected cells. Further, we detected DCs which are critical for antigen presenting and priming T cells,^36^ decline in number within higher age range. Potentially a DCs reduction contributes to an age-related shift in T cell profiles from a naive toward effector memory phenotype. Women demonstrated an age × sex interaction in MAIT/NKT counts, decreasing more sharply in older age (50–93 years). In contrast, men exhibited higher invariant T cell counts in later life. These specialized T cell subsets are vital for combating diverse pathogens i.e., *Staphylococcus aureus*,^4^^,^^26^^,^^43^ and their reduced numbers in older women might increase vulnerability to chronic infections.^44^ Our findings accentuate distinct sex-specific shifts in immune cell composition, which interact with age-related differences in immune cell subpopulations. This suggests that immunological aging is likely a complex process influenced by an interplay between chronological age and biological sex^33^ and also environmental factors such as infections etc. In our study population, we observed five T cell subpopulations in elderly individuals, which correlated with a reduction in CD28 intensity in some of these populations compared to younger adults (data not shown). These findings emphasize the potential role of senescence markers in understanding the intricate mechanisms underlying immune aging, but this should be further explored using senescence-associated markers.

Alterations in B cell populations aligned with an age × sex interaction for B_mem_ cell counts in women, demonstrate a more pronounced decrease between ages 20–56. Women lose naive B cells a decade later than men (women: 64–93 years, men: 52–93 years). Notably, the naive B cell subset increased in women but decreased in men. From middle age onward, women consistently had higher mobilized naive B cell counts than men (age 49–74), potentially explaining the earlier onset of pneumonia in men and the decreased efficacy of the T-independent polysaccharide pneumococcal vaccine in elderly men.^22^ To the best of our knowledge, this is the first study that discovered the age × sex interaction within the B_mem_ cell population. The observed accelerated reduction in B_mem_ cells in women may correspond with the timing of naive B cell retention, implying a dynamic compositional balance in B cell subpopulations over lifespan. We observed a higher frequency of follicular B cells in premenopausal females compared to older females and age-matched males. Then we followed age and sex interaction in this particular B cell population, in which we observed that while males maintain a relatively stable frequency of these cells throughout life, females exhibit a continuous decline from approximately age 27 toward later lifetime (93 years old). This difference can likely be attributed to the influence of sex hormones on the immune system. During the transition into menopause, declining estrogen levels may contribute to a reduction in B cell numbers, highlighting the potential role of hormonal regulation in shaping immune cell populations.^45^ This observation raises the question of whether declining follicular B cell frequency contribute to the age-related changes in humoral immunity in females, and warrants further investigation. Naive CD27^−^ B cells have the competence to differentiate into long-lived plasma cells,^46^ which could explain the superior humoral immunity opined in women, as evidenced by higher antibody responses to various pathogens and higher basal immunoglobulin levels.^47^

Chronic inflammation in the elderly impacts health and contributes to immune senescence, fostering age-related pathologies.^33^ Monocytes are key players in innate immune defense.^7^ We found that adult men have higher transitional monocyte counts than women of the same age at 44–78. However, we observed no age × sex interaction in monocyte subpopulations within either sex. As transitional monocytes bridge the gap between classical and non-classical subsets with distinct functional properties,^25^ elevated levels in men suggest a potentially heightened inflammatory state.

Granulocytes as a part of the innate immune system become less functional and paradoxically exhibit a propensity for heightened basal inflammation through increased secretion of inflammatory proteins i.e., granzymes and cytokines with age.^2^ However, despite the fact we have not analyzed the functionality of granulocytes over lifetime, our finding showed that the counts of granulocytes (i.e., neutrophils) obtained from individuals are stable over lifespan, and we have not observed age- and sex-related effects in none-granulocyte subpopulation.

In conclusion, our findings showed an interplay between age and sex in influencing immune cell phenotype composition by a different statistical approach. A deeper understanding of the complex interplay between age and sex in immune-aging will facilitate the exploration of their distinct impacts on immune responses, potentially improving health outcomes across the lifespan and informing future clinical studies to consider the sex- and age-specific effects on immune cell composition.

### Limitations of the study

While this study provides insights into the compositional variation of blood circulating immune cells between sex at different ages, our study has several limitations. One limitation is the low number of cases in some age ranges (e.g., low numbers of persons around age 20 and older persons >90). Each observation leads to imprecise estimates of the regression curves in these age ranges (e.g., wide confidence limits) but does not imply biased model estimation. Also, it should be noted that the overarching question of our study concerns the aging of the immune system over the lifespan. Therefore, our analyses are limited to the use of cross-sectional data, which describe interindividual instead of intraindividual differences over time. Additional assumptions are required to interpret the relationship between age and the cross-sectional cytometric data as change. The key assumption is that the stochastic relationship between age and cell counts is invariant across all birth cohorts, both in the total population and within the two sex groups. This is an untestable assumption in our sample, which needs to be kept in mind when interpreting the results.

In addition, the study is restricted to one village in rural Thuringia only. Therefore, an analysis of other cohorts might improve the external validity of our findings. In the current state of study, not implementing functionality assay for immune cell populations, preventing us from correlating our findings with the functional status of these immune cells. Furthermore, future investigations should delve into the specific hormonal changes occurring during different age ranges to comprehensively understand the impact of menopause on immune cell composition, an aspect not addressed in this cohort study. Additionally, the focus is solely on circulating blood immune cells. Potentially, this risks to overlook a possible compensatory role of other tissue resident lymphatic cells.

## Resource availability

### Lead contact

Further information and requests for resources and reagents should be directed to and will be pfulfilled by the lead contact, Mathias W. Pletz (mathias.pletz@med.uni-jena.de).

### Materials availability

All unique materials generated in this study are available from the lead contact upon request.

### Data and code availability

• Data: All data supporting the findings of this study are available within the paper and its supplemental information files.
• Code: The analysis scripts used in this study are available from the lead contact upon reasonable request. Specific software versions and packages used are detailed in the STAR Methods section.
• Other: Any additional information required to reanalyze the data or replicate the study is available from the lead contact upon reasonable request.

## Acknowledgments

We would like to thank the technical staff of the institute of Immunology, Jena University Hospital, Friedrich-Schiller University for their support in this study.

CoNAN study group: the project was conducted in cooperation with the district administration and the health department of the Ilm district. Local cooperation partners: Petra Enders, Renate Koch, Steffen Mai, Matthias Ullrich, Dagmar Rimek. Institute of Clinical Chemistry and Laboratory Diagnostics and Integrated Biobank Jena (IBBJ), Jena University Hospital, Friedrich-Schiller-University, Jena, Germany: Cora Richert, Cornelius Eibner, Bettina Meinung, Kay Stötzer, Julia Köhler. Children’s Hospital, Jena University Hospital, Friedrich-Schiller-University, Jena, Germany: Hans Proquitté, Hans Cipowicz, Christine Pinkwart. Department of Anesthesiology and Intensive Care Medicine Jena University Hospital—Friedrich Schiller University, Jena, Germany: Michael Bauer, Petra Dickmann, Annika Licht, Juliane Scholz, Wibke Wetzker. Institute for Infectious Disease and Infection Control, Jena University Hospital, Friedrich-Schiller-University, Jena, Germany: Gabi Hanf, Jasmin Müller, Jennifer Kosenkow, Franziska Röstel, Juliane Ankert, Aurelia Kimmig, Stefan Hagel, Christina Forstner. Institute of Immunology, Jena University Hospital, Friedrich-Schiller-University, Jena, Germany: Raphaela Marquardt. Institute of Medical Microbiology, Jena University Hospital, Friedrich-Schiller-University, Jena, Germany: Sebastian Kuhn.

The CoNAN study was funded by the Sondervermögen “Corona” of the Thuringian Ministry for Economic Affairs, Science and Digital Society (TMWWDG), SV-Kapitel 82 30 Titel 68205 # 5526/32-4-2. Further support was by the 10.13039/501100002347Federal Ministry of Education and Research grant no: 01KX2121 and 13N15745.

Role of theSponsor: The funding agencies had no role in the design and conduct of the study; collection, management, analyses, and interpretation of the data; preparation, review, or approval of the manuscript; and decision to submit the manuscript for publication.

## Author contributions

Conceptualization: M.W.P., S.W., and T.K.; methodology: S.B., N.A., and R.G.; investigation: S.B., N.A., R.G., and D.R.; formal analysis: N.R., S.B., R.G., and N.A.; resources: M.W.P., T.K., S.W., and O.M.; data curation: R.G., D.R., S.D., and C.S.; writing – original draft preparation: R.G. and S.B.; writing – review and editing: all authors; visualization: R.G., N.R., S.B., and S.W.; supervision and project administration: M.W.P., S.W., and O.M.

## Declaration of interests

All authors declare no competing interests.

## STAR★Methods

### Key resources table

**Table:** 

REAGENT or RESOURCE	SOURCE	IDENTIFIER
**Antibodies**		

CD45 (Clone HI30, Isotope^89^Y)	Standard BioTools Inc	RRID: AB_2810854
CD196/CCR6 (G034E3,^141^Pr)	Standard BioTools Inc	RRID: AB_2687639
CD123 (6H6,^143^ND)	Standard BioTools Inc	RRID: AB_2661794
CD19 (HIB19,^144^ND)	Standard BioTools Inc	RRID: AB_2893034
CD4 (RPA-T4,^145^ND)	Standard BioTools Inc	RRID: AB_3661845
CD8a (RPA-T8,^146^ND)	Standard BioTools Inc	RRID: AB_2811089
CD11c (Bu15,^147^Sm)	Standard BioTools Inc	RRID: AB_2687850
CD16 (3G8,^148^ND)	Standard BioTools Inc	RRID: AB_3665424
CD45RO (UCHL1,^149^Sm)	Standard BioTools Inc	RRID: AB_2687851
CD45RA (HI100,^150^ND)	Standard BioTools Inc	RRID: AB_3677849
CD161 (GP-3G10,^151^Eu)	Standard BioTools Inc	RRID: AB_2687651
CD194/CCR4 (L291H4,^152^Sm)	Standard BioTools Inc	RRID: AB_2687647
HLA-DR (LN3,^173^Yb)	Standard BioTools Inc	RRID: AB_2810248
CD25 (BC96,^153^Eu)	Standard BioTools Inc	RRID: AB_3677869
IgD (IA6-2,^174^Yb)	Standard BioTools Inc	RRID: AB_2811082
CD127 (A019D5,^176^Yb)	Standard BioTools Inc	RRID: AB_3665122
CD27 (O323,^154^Sm)	Standard BioTools Inc	RRID: AB_3094744
CD57 (HCD57,^155^Gd)	Standard BioTools Inc	RRID: AB_2756434
CD183/CXCR3 (G25H7,^156^Gd)	Standard BioTools Inc	RRID: AB_2810969
CD185/CXCR5 (J252D,^158^Gd)	Standard BioTools Inc	RRID: AB_2858239
CD28 (CD28.2,^160^Gd)	Standard BioTools Inc	RRID: AB_2868400
CD38 (HB-7,^161^Dy)	Standard BioTools Inc	RRID: AB_3677930
CD56/NCAM (NCAM16.2,^163^Dy)	Standard BioTools Inc	RRID: AB_2938638
TCRgd (B1,^164^Dy)	Standard BioTools Inc	RRID: AB_2687643
CD294 (BM16,^166^Er)	Standard BioTools Inc	RRID: AB_2810253
CD197/CCR7 (G043H7,^167^Er)	Standard BioTools Inc	RRID: AB_2858236
CD14 (63D3,^168^Er)	Standard BioTools Inc	RRID: AB_2687634
CD3 (UCHT1,^170^Er)	Standard BioTools Inc	RRID: AB_2661807
CD20 (2H7,^171^Yb)	Standard BioTools Inc	RRID: AB_2802112
CD66b (G10F5,^172^Yb)	Standard BioTools Inc	RRID: AB_3677863
Live/dead –DNA intercalator (N/A,^103^Rh^191^Ir/^193^Ir)	Standard BioTools Inc	N/A

**Chemicals, peptides, and recombinant proteins**		

Heparin	Sigma Aldrich	Cat #H3149-25KU
Cal-Lyse™ Lysing Solution	Thermo Fisher Scientific	Cat #GAS010
Paraformaldehyde	Electron Microscopy Sciences	Cat #157-4-1L

**Critical commercial assays**		

Maxpar® Direct™ Immune Profiling Assay™	Standard BioTools Inc	Cat #201325.
Trucount™ Beads	BD Biosciences	Cat #663028
Maxpar® Fix and Perm Buffer	Standard BioTools Inc	Cat #201067

**Software and algorithms**		

Maxpar Pathsetter	Standard BioTools Inc	V 3.0

### Experimental model and study participant details

The CoNAN study (*COVID-19 Outbreak in Neustadt-am-Rennsteig*) was initiated after a SARS-CoV-2 outbreak in Spring 2020 in Neustadt am Rennsteig (Thuringia, Germany) focusing to investigate the seroprevalence and potential development of immunity of SARS-CoV-2 infections (detailed information in Schnizer et al.^21^). All participants of the present study include 117 women and 114 men. Peripheral blood was taken at two time points, in May or October 2020, respectively. Only subjects with seronegative and negative PCR test against SARS-Cov-2 (<4 out of six different tests) at the earlier time point (May 2020), and subjects with no symptoms for respiratory infects at the later time point (October 2020) were enrolled in this study. Considered exclusion criteria were based on the CoNAN cohort, and all acutely ill subjects were not enrolled in this study. The CoNAN study protocol was reviewed and approved by the institutional ethics committees of the Jena University Hospital and the respective data protection commissioner (approval number 2020-1776) and the ethics committee of the Thuringian chamber of physicians. The study was conducted according to the current version of the Declaration of Helsinki. All data were collected with unique pseudonyms on paper case report forms. These identifiers were later used to merge the questionnaire information with the laboratory information in an electronic study database. Using cubic smoothing spline regression, which does not make specific assumptions about how the cell counts change over time. Instead of conventional parameters, such as regression coefficients of independent variables or polynomial terms in common parametric regression models, our statistical inference is based on the first derivatives of the nonparametric regression curves, which reflects the rate at which cell counts are increasing or decreasing at any given age (see Figure S1). A positive rate indicates an increase in cell counts, while a negative rate indicates a decrease. We used the nonparametric bootstrap method to account for sampling error in the derived quantities of the regression curves (e.g., predicted values and first derivatives), which involves generating numerous simulated datasets (see Figures S2). This method allowed us to calculate 95% confidence bands, representing the range within which we are 95% certain the true predicted values or true rates of change lie. We performed this process 2,000 times for each type of immune cell.

### Method details

Heparinized whole blood was used to assess the absolute number of leukocytes by using Trucount Beads (BD Biosciences, San Jose, CA, USA) and flow cytometry (BD FACSCanto Plus Cell Analyzer). For high dimensional immune profiling of peripheral blood leukocytes, a commercially available 30-marker antibody panel was used (Maxpar Direct Immune Profiling System, Standard BioTools, South San Francisco, CA, USA).^3^ Briefly, 270 μL of whole blood was incubated with heparin (H3149-25KU, Sigma Aldrich) as blocking reagent (30 μL of 1,000 U/mL heparin in PBS), incubation at room temperature (RT), 20 min and then incubated for 30 min at RT with lyophilized antibody cocktail. Red blood cells were lysed using 250 μL CAL-Lyse Lysing Solution (Life Technologies, Rockville, MD, USA) (incubation at RT, 10 min, dark). Three mL Millipore Q water was added and further incubated (RT, 10 min, dark). After centrifugation (300 g) for 5 min and washing the cells three times with MaxPar Cell staining buffer, cell pellet was resuspended and fixed with 1 mL 1.6 paraformaldehyde (EMS, Hatfield, PA, USA) (RT, 10 min). After centrifugation (800 g) cells were incubated with 1 mL Maxpar Fix and Perm Buffer with 1:1,000 iridium (Cell-ID DNA intercalator, 125 μM (4°C, 2 h)). Cells were centrifuged (800 g) and 900 μL supernatant were discarded. The remaining was stored at −80°C until mass cytometric measurement (CyTOF, Cytometry time of flight).

Cells were thawed, washed two times with 2 mL MaxPar Cell staining buffer, and an additional two times with Cell acquisition solution. Cells were counted using a hemacytometer and adjusted to 7.5 x10^5^ cells/mL. 10% v/v Four Element calibration beads (Standard BioTools) as internal standard beads were added before acquisition of an average of 500,000 events using the Helios mass cytometer equipped with CyTOF software v7.0.8496.0 (Standard BioTools). FCS data files were normalized based on signals of the internal standard beads.

### Quantification and statistical analysis

Mass cytometry (CyTOF) data analysis: Pre-cleaning (removing debris, dead cells and doublets) as well as data analysis were performed automatically using MaxPar Pathsetter software 2.x to obtain frequencies of 35 leukocytic cell subsets, which were converted into absolute counts for subsequent statistical analysis for immune-aging (Table S3).

For in-depth immunophenotyping of T- and B-lymphocyte compositional differences, cleaned data were pre-gated on T-cells (CD45^+^CD66b^−^CD19^−^CD14^−^CD3^+^) and B-cells (CD45^+^CD66b^−^CD3^−^CD14^−^CD56^−^CD19^+^) using FlowJo v10.8.1 (BD Biosciences, San Jose, CA, USA). New generated FCS files were further analyzed and visualized with OMIQ (Omiq, Boston, MA, USA).^23^ Uniform Manifold Approximation and Projection (UMAP) as dimensionality-reduction visualization tool and FlowSOM (self-organization maps) as an automated clustering algorithm. The statistical analysis of cell cluster frequencies were performed in GraphPad Prism 9 (GraphPad Software, San Diego, California, USA). The One-Way-ANOVA was applied and *p*-values less than 0.05 were considered significant.

For statistical analysis of immune cell counts in different ages, we used cubic smoothing spline regressions (SSR). As SSR models do not have any testable parameters, we decided to use 95% confidence interval bands (95%-CB) for the first derivatives $f^{\prime }\left(Age\right)=\frac{d}{d\;Age}\;E\left(Y\;|\;Age\right)$ of the spline regression *E*(*Y* | *Age*) as an alternative for identifying age ranges with significant changes in the cell counts. The first derivatives of non-linear regression curve at any *Age* = *x* is the slope of tangent line of that curve, which provides an estimate of the instantaneous rate of change at *Age* = *x*. If *f’* (*Age* = *x*) > 0 there is an increase in the cell counts while negative values *f’*(*Age* = *x*) < 0 indicate declining cell counts at any *Age* = *x*. Age ranges in which the 95% confidence band does not overlap zero show statistically significant changes in cell counts. The 95%-CB were obtained via nonparametric bootstrap with 2,000 bootstrap samples for each immune cell type (Figure S1).

The interaction effect *Age × Sex* regarding the immune cell counts refers to sex differences in the change rate in certain age ranges. If Let *E*_*femal*_(*Y* | *Age*) the SSR of cell counts *Y* on *Age* for women and *E*_*male*_(*Y* | *Age*) for men, with the first derivatives $f_{female}^{\prime }\left(Age\right)=\frac{d}{d\;Age}\;E_{female}\left(Y\;|\;Age\right)$ and $f_{male}^{\prime }\left(Age\right)=\frac{d}{d\;Age}\;E_{male}\left(Y\;|\;Age\right)$. Using SSR models, an interaction effect means a difference in the instantaneous change rate at any Age = x, which is formally the difference $f_{female}^{\prime }\left(Age\right)-f_{male}^{\prime }\left(Age\right)$ of the first derivatives of the two sex-specific regression curves. Age ranges in which the 95% confidence band of the difference $f_{female}^{\prime }\left(Age\right)-f_{male}^{\prime }\left(Age\right)$ does not overlap zero show statistically significant interaction effects between age and sex. Nonparametric bootstrap was used to obtain the 95%-CBs of the first derivatives $f_{female}^{\prime }\left(Age\right)$ and $f_{male}^{\prime }\left(Age\right)$ as well as the difference $f_{female}^{\prime }\left(Age\right)-f_{male}^{\prime }\left(Age\right)$, with 2,000 bootstrap samples per each immune cell type (Figure S2).

All models were fitted in R with the ‘smooth.spline’ function for fitting the SSR, and ‘predict.smooth.spline’ function for computing the first derivatives of the regression curves (50). Due to quite wiggly regression curves in the bootstrap samples and to avoid overfit, we opted for more conservative values of the smoothness parameter λ than suggested by the cross validation. This was achieved by specifying the scale free tuning parameter spar = 0.8.

### Additional resources

All donors included in the present study were participants of the CoNAN-Study with German Clinical Trials Register number: DRKS00022416.
