# Supplementary material for: Age- and sex-associated differences in immune cell populations
Source: iScience. 2025 Jul 10;28(8):113092. doi: 10.1016/j.isci.2025.113092 (PMC12309924; doi:10.1016/j.isci.2025.113092)
Supplement: Document S1. Figures S1–S5 and Tables S1–S6 [file mmc1.pdf]

iScience, Volume *28*

Supplemental information

Age- and sex-associated differences in immune cell populations

Reza Gheitasi, Sabine Baumgart, Daniela Roell, Norman Rose, Carsten Watzl, Diana Dudziak, Nico Andreas, Oliwia Makarewicz, Sebastian Drube, Clara Schnizer, Thomas Kamradt, Sebastian Weis, Mathias W. Pletz, and for the CoNAN study group

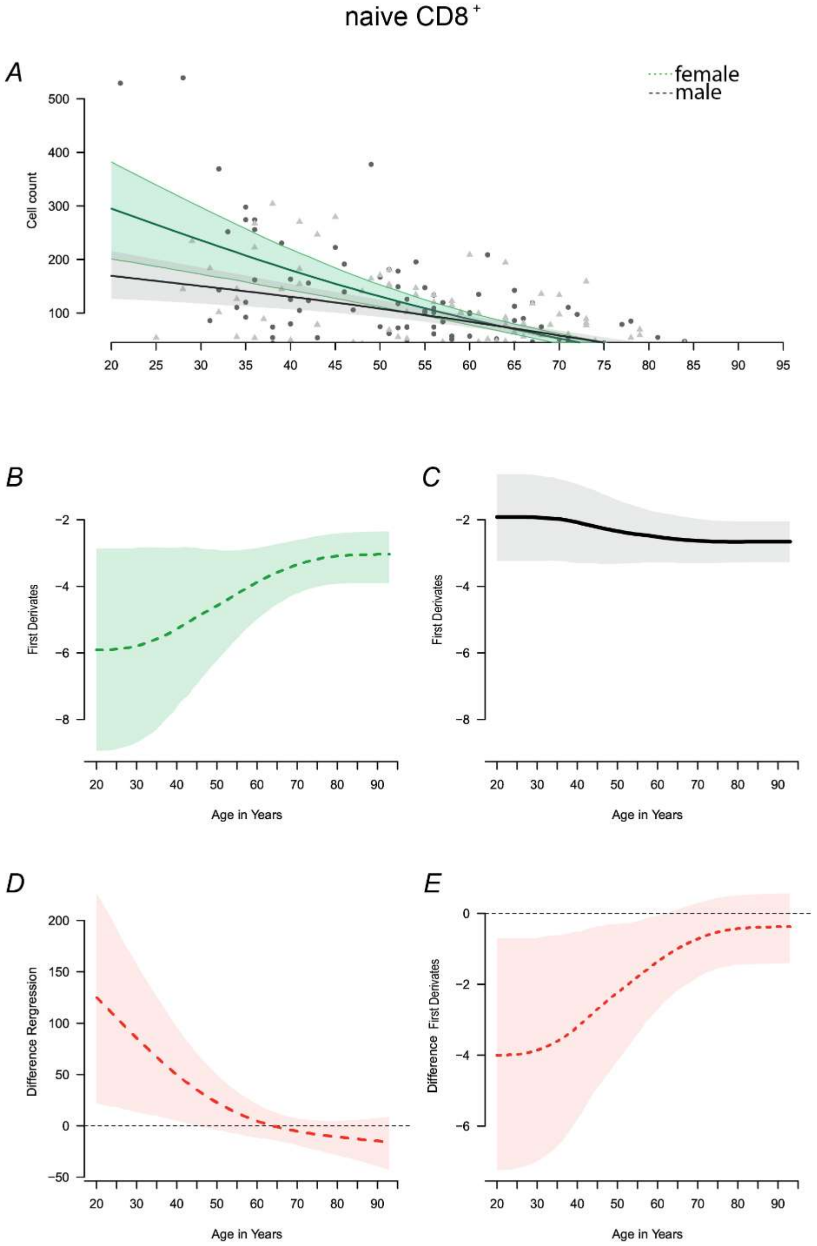

Figure S1: Spline regression of naïve CD8^{+} T-cell counts: The spline regression approach was utilized for analyzing (i) differences in cell counts between sexes at different ages, and (ii) the interaction between age and sex, **(A)** Sex-specific regression curves at each age with 95% confidence bands, respectively. **(B and C):** Slopes (first derivative) of individual regression curves (females (B) and males (C)): Hence, the slope is more negative in women than in men over the whole age range for naïve CD8^{+} T-cell counts. **(D)** Differences of the sex-specific regression curves at each age with the 95% confidence band. Statistically significant differences between both sexes at a particular age are inferred from the regression analysis if the difference

between the two sex specific regression curves at this age are different from zero. This is the case if the 95% confidence intervals of the differences of the regression curves (e.g., the difference of the expected values of cell counts for male and female at each age) do not overlap with zero at the considered age. Hence, significantly higher numbers of naïve $CD8^+$ T-cells in females are found in the age of 20 and 44. The decline of the mean differences in this age range is due to the stronger decline (e.g., more negative slope) in naive $CD8^+$ T-cells in females compared to males. **(E)** Difference of the slopes of the sex-specific regression curves at each age with the 95% confidence band: In general, differences in the slopes of the sex-specific regression curves indicate the interaction between age and sex. Statistically significant differences of the slopes and, therefore, a significant interaction effect is indicated by 95% confidence intervals that do not overlap with zero. Hence, the interaction effect regarding naïve $CD8^+$ T-cells can be seen in the stronger decline of cell counts in women compared to men in the age of 20 and 62.

Abbreviations: FirstDerivate: first derivative

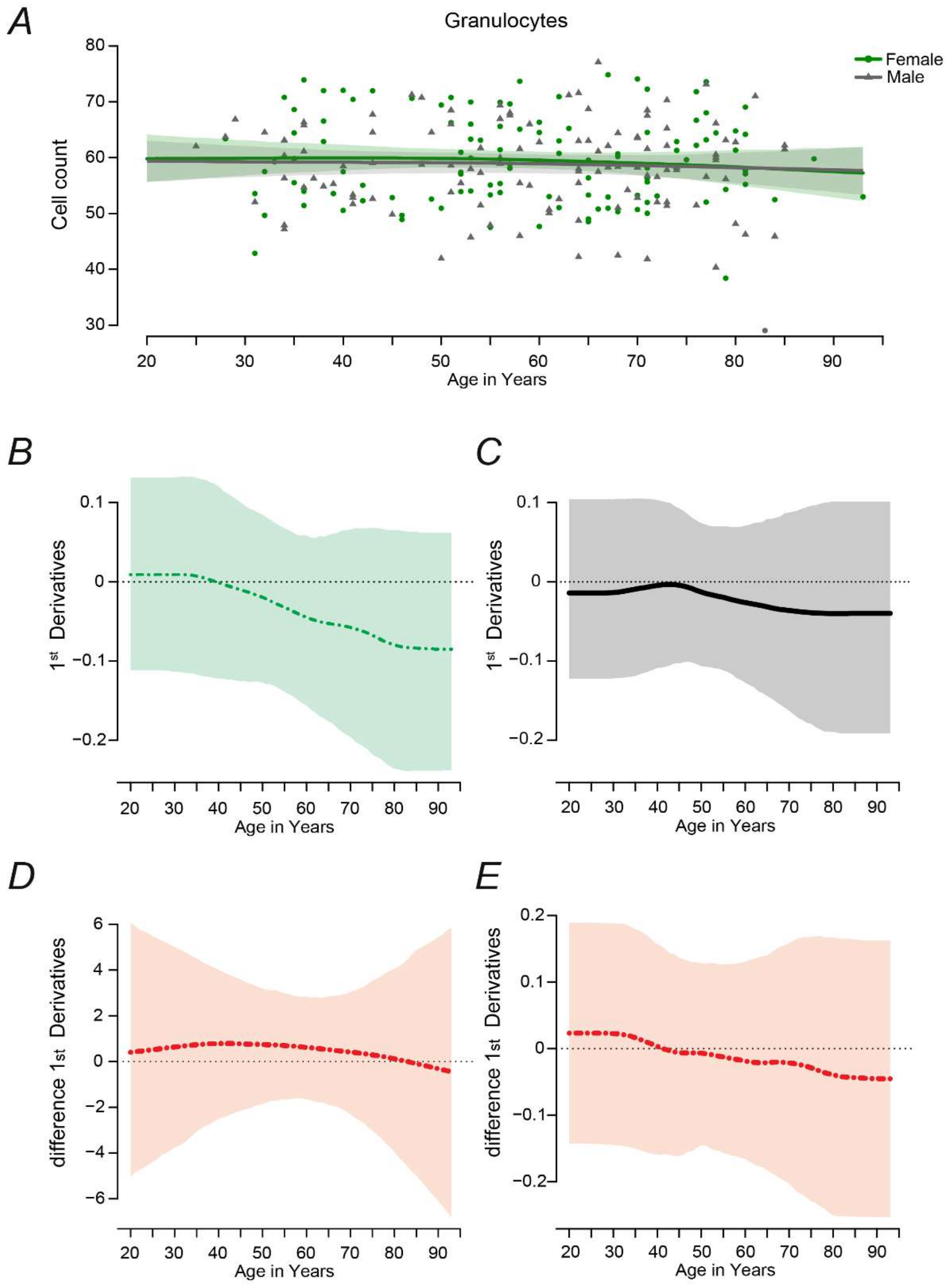

Figure S2: Sex-specific smoothing spline regressions of cell count of granulocytes on age, its first derivative, and the interaction age sex. **(A)** The scatterplot of age and cell counts including the two sex-specific regression curves. **(B-C)** first derivative of regression curves of females (B) and males (C). **(D)** Difference of the two sex-specific regression curves with the 95% confidence band. **(E)** Difference of the first derivative of the two sex-specific regression curves with the 95% confidence band (age and sex interaction effect).

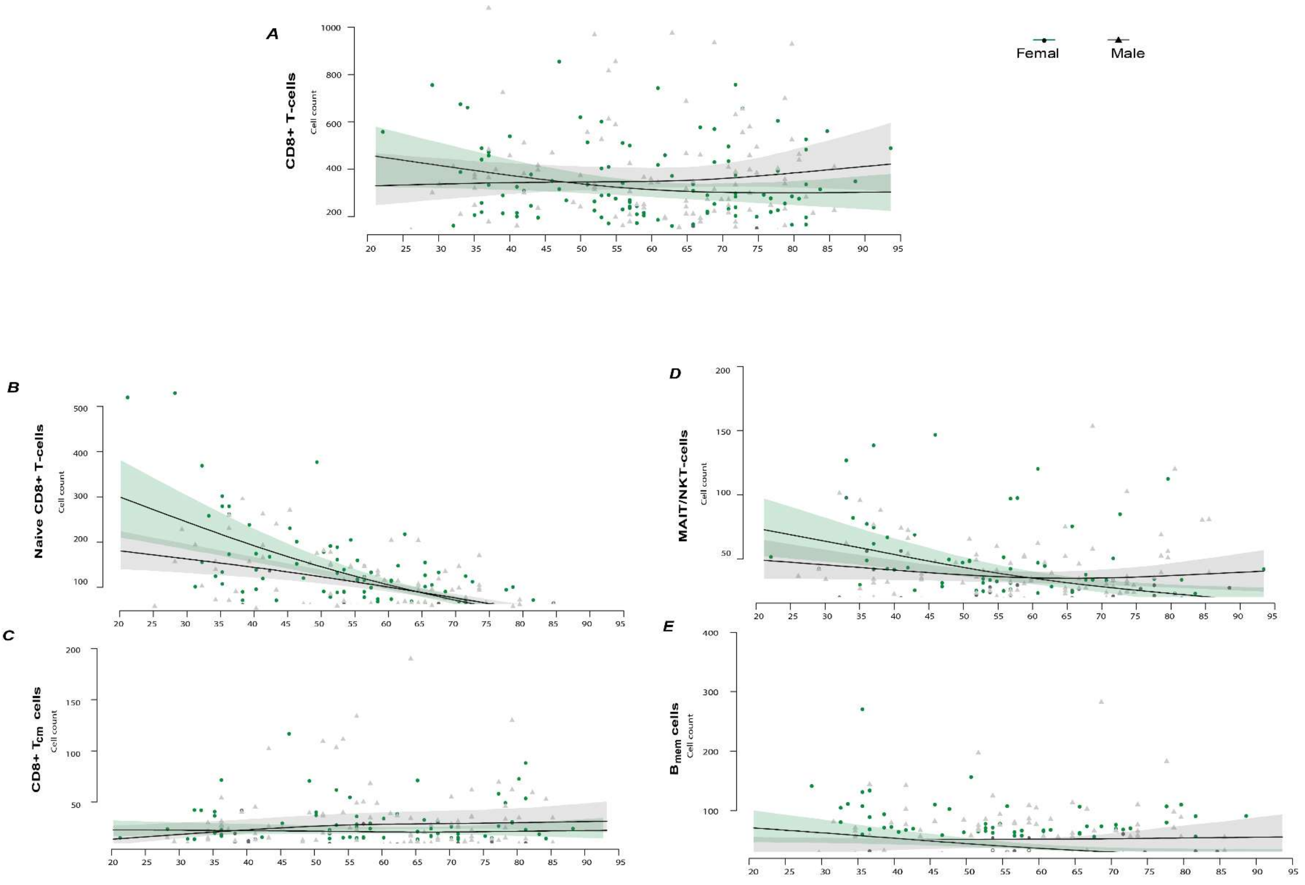

Figure S3: Spline regression of absolute cell number of **(A)** CD8^{+} T-cells, **(B)** Naïve CD8^{+} T-cells, (C) CD8^{+} T_{cm} cells, **(D)** MAIT/NKT-cells, **(E)** B_{mem} cells. The spline regression approach was utilized for analyzing (i) differences in cell counts between sexes at different ages, and (ii) the interaction between age and sex, (A) Sex-specific regression curves at each age with 95% confidence bands, respectively.

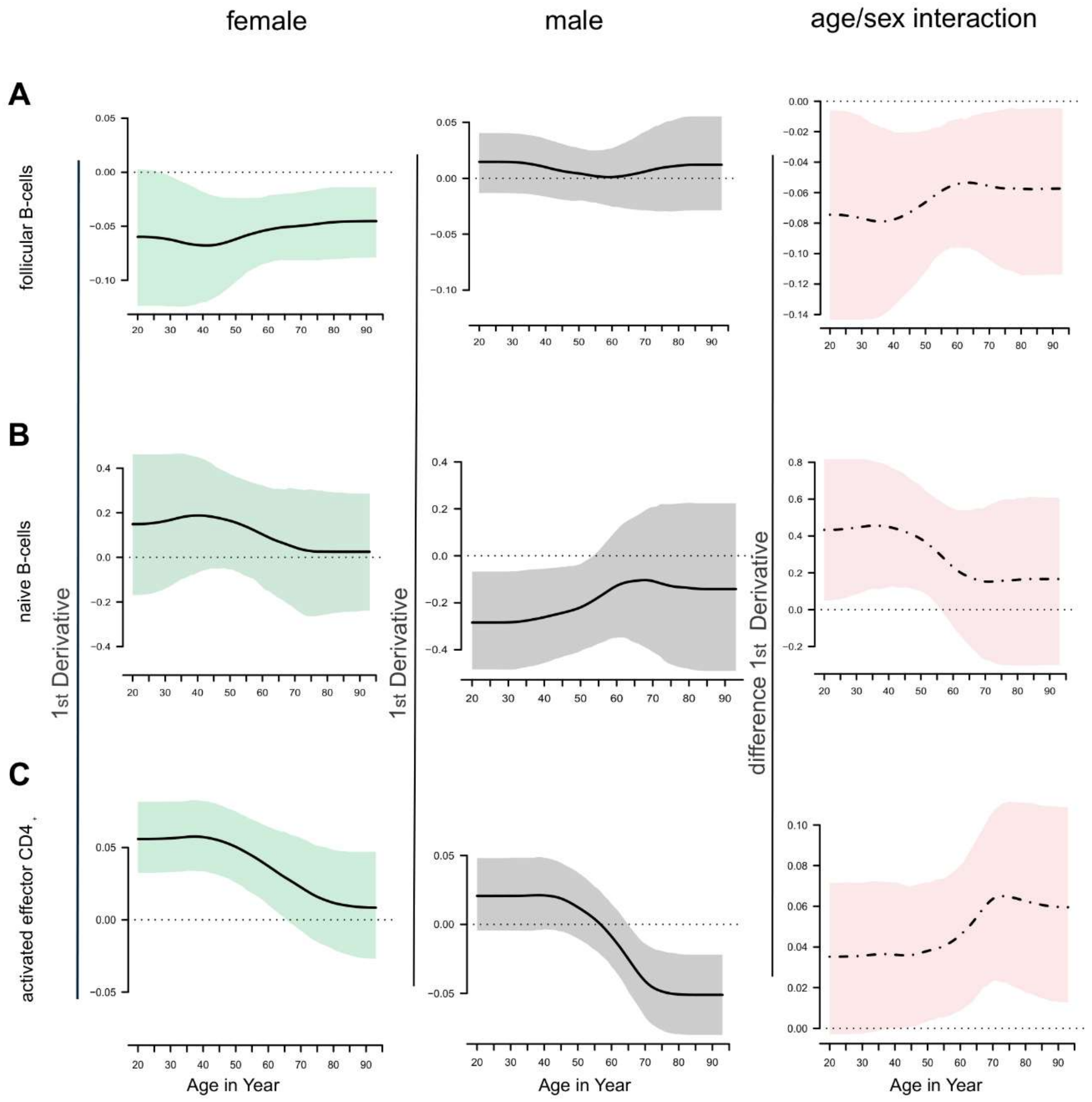

Figure S4 First derivatives of sex-specific spline regressions of cell counts (left and middle column) and age × sex interaction effect (right column) representing the difference of the first derivative of the two sex-specific regression curves with 95%-confidence band, respectively, for **(A)** follicular B-cells, **(B)** naïve B-cells, **(C)** activated effector $CD4^+$ T-cells in regard to age.

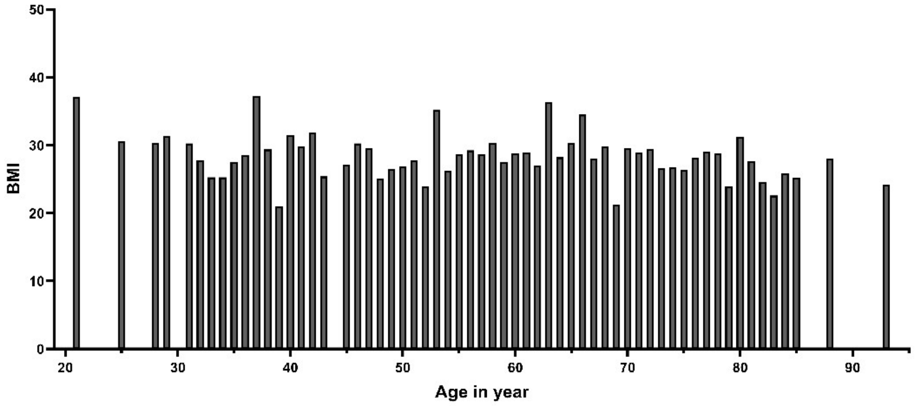

Figure S5: Body mass index (BMI) distribution of study participants across age. **A)** The x-axis represents age in years, and the y-axis displays the BMI of the participants.

Table S1: Demographic characteristics of individuals participated in the study.

	**Female (n=117)**	**Male (n= 114)**
Age (mean, (SD))	58 (16)	59 (15)
Age (median (min, max))	58 (21, 93)	62 (25, 85)
BMI (mean, (SD))	28 (6)	29 (5)
BMI (median (min, max))	28 (18, 44)	28 (19, 49)
Arterial hypertension (n, percent)	50 (43 %)	61 (54 %)
Myocardial infarction (n, percent)	4 (3 %)	9 (8 %)
Congestive hearte failure (CHD) (n, percent)	18 (16 %)	14 (12 %)
Peripheral arterial disease(pAVK) (n, percent)	10 (9 %)	2 (2 %)
Stroke (n, percent)	2 (2 %)	6 (5 %)
Chronic lung disease (n, percent)	8 (7 %)	8 (7 %)
Autoimmune disease/immunodeficiency (n, percent)	10 (9 %)	12 (11 %)
Liver disease (n, percent)	2 (2 %)	21 (18 %)
Diabetes mellitus (n, percent)	9 (8 %)	32 (28 %)
Chronic renal disease (n, percent)	15 (13 %)	22 (19 %)
Tumor (n, percent)	5 (4 %)	4 (4 %)
Chronic wounds, eczema (n, percent)	4 (3 %)	5 (4 %)
Chronic viral infection (n, percent)	0 (0 %)	1 (1 %)
Other disease (n, percent)	32 (28 %)	17 (15 %)
Smoker (n, percent)	51 (47 %)	49 (43 %)
Former Smoker (n, percent)	8 (7 %)	13 (11 %)
Congestive hearte failure, CHD (n, percent)	18 (16 %)	14 (12 %)
pAVK (n, percent)	10 (9 %)	2 (2 %)
Chronic lung disease (n, percent)	8 (7 %)	8 (7 %)

*n = number, *percent = percentage, *SD = standard deviation, *min = minimum, *max = maximum.

Table S2: Phenotypic characterization of leukocyte lymphocytic lineage used for smooth spline regression analysis.

**Populations**	**Phenotypes**
Lymphocytes	CD3 T-cells + B-cells + NK cells + plasmablasts
CD3^{+} T-cells	CD8^{+} T-cells + CD4^{+} T-cells + γδ T-cells + MAIT/NKT-cells
CD8^{+} T-cells	CD3^{+} CD66b^{-} CD19^{-} CD8^{+} CD4^{-} CD14^{-} CD161^{-} TCRgd^{-} CD123^{-} CD11c^{-}
CD8 naïve	CD8 T-cells + CD45RA^{+} CCR7^{+} CD27^{+}
CD8^{+} T_{cm} cells	CD8 T-cells + CD45RA^{-} CCR7^{+} CD27^{+}
CD8^{+} T_{em} cells	CD8 T-cells + CCR7^{-} CD27^{+}
CD8^{+} TEMRA	CD8 T-cells + CCR7^{-} CD27^{-}
CD4^{+} T-cells	CD66b^{-} CD3^{+} CD8^{-} CD4^{+} CD14^{-} TCRgd^{-} CD11c^{-}
CD4^{+} naïve	CD4 T-cells + CD45RA^{+} CCR7^{+} CD27^{+}
CD4^{+} T_{cm} cells	CD4 T-cells + CD45RA^{-} CCR7^{+} CD27^{+}
CD4^{+} T_{em} cells	CD4 T-cells + CD45RA^{-} CCR7^{-} CD27^{+}
CD4^{+} TEMRA	CD4 T-cells + CD45RA^{-} CCR7^{-} CD27^{-}
T_{reg} cells	CD4 T-cells + CD25^{+} CD127^{-} CCR4^{+}
Th1-like	CD4 T-cells + CXCR3^{+} CCR6^{-} CXCR5^{-} CCR4^{-}
Th2-like	CD4 T-cells + CXCR3^{-} CCR6^{-} CXCR5^{-} CCR4^{+}
Th17-like	CD4 T-cells + CXCR3^{-} CCR6^{+} CXCR5^{-} CCR4^{+}
γ T-cells	CD66b^{-} CD3^{+} CD8$^{dim,-}$ CD4^{-} CD14^{-} TCRgd $^{dim,+}$
MAIT/NKT-cells	CD66b^{-} CD3^{+} CD4^{-} CD14^{-} CD161^{+} TCRgd^{-} CD28^{+} CD16^{-}
B-cells	CD3^{-} CD14^{-} CD56^{-} CD16 $^{dim,-}$ CD19^{+} CD20^{+} HLA-DR $^{dim,+}$
B naïve	B-cells + CD27^{-}
B_{mem} cells	B-cells + CD27^{+}
Plasmablasts	CD3^{-} CD14^{-} CD16$^{-,dim}$ CD66b^{-} CD20^{-} CD19^{+} CD56^{-} CD38^{++} CD27^{+}
NK cells	CD14^{-} CD3^{-} CD123^{-} CD66b^{-} CD45RA^{+} CD56 $^{dim,+}$
NK early	NK cells + CD57^{-}

NK late	NK cells + $CD57^{+}$
Monocytes	$CD3^{-}$ $CD19^{-}$ $CD56^{-}$ $CD66b^{-}$ $HLA\text{-}DR^{+}$ $CD11c^{+}$
Monocytes classical	Monocytes + $CD14^{+}$ $CD38^{+}$
Monocytes transitional	Monocytes + CD14 ^{dim} CD38 ^{dim}
Monocytes non-classical	Monocytes + $CD14^{-}$ $CD38^{-}$
pDCs	$CD3^{-}$ $CD19^{-}$ $CD14^{-}$ $CD20^{-}$ $CD66b^{-}$ HLA-DR $^{dim,+}$ $CD11c^{-}$ $CD123^{+}$
cDCs	$CD3^{-}$ $CD19^{-}$ $CD14^{-}$ $CD20^{-}$ HLA-DR $^{dim,+}$ CD11c $^{dim,+}$ $CD123^{-}$ CD16 $^{dim,-}$ CD38 $^{dim,+}$ $CD294^{-}$ HLA-D
Granulocytes	Neutrophils + basophils + eosinophils + $CD66b^{-}$ neutrophils
Neutrophils	CD66b $^{dim,+}$ $CD16^{+}$ $HLA\text{-}DR^{-}$
Basophils	$HLA\text{-}DR^{-}$ $CD66b^{-}$ CD123 $^{dim,+}$ $CD38^{+}$ $CD294^{+}$
Eosinophils	$CD14^{-}$ $CD3^{-}$ $CD19^{-}$ $HLA\text{-}DR^{-}$ $CD294^{+}$ CD66b $^{dim,+}$
CD66b- neutrophils	$CD3^{-}$ $CD19^{-}$ $CD66b^{-}$ $CD56^{-}$ $HLA\text{-}DR^{-}$ $CD123^{-}$ $CD45^{-}$

Abbreviations: pDC: plasmacytoid dendritic cells, cDCs: conventional dendritic cells, NK: Natural killer cells, T_{em}: Effector memory, TEMRA: terminally differentiated effector memory RA+, T_{cm}: Central memory, MAIT/NKT: mucosa-associated invariant T-cells/ Natural Killer T-cells.

Table S3: Significant changes of immune cell counts of all subjects included in this study regardless of sex (age ranges with zero outside of the 95%-confidence band of the first derivative of the smoothing spline regressions) .

Immune Cell type	Decrease	Increase
Transitional Monocyte	-	63-64*
Non-classic Monocyte	-	20-57
Total dendritic cells	73-93	-
Plasmacytoid DC	75-93	-
Myeloid DC	81-93	-
Early NK cells	-	30-33; 63-66
Late NK cells	-	30-35; 63-66
Total $CD8^+$ T-cells	53-55	-
Naive $CD8^+$ T-cells	38-93	-
CM $CD8^+$ T-cells	-	20-34
EM $CD8^+$ T-cells	53-55	-
TE $CD8^+$ T-cell	-	20-36; 61-74
Total $CD4^+$ T-cells	58-63; 69-75	-
Naive $CD4^+$ T-cells	68-93	-
EM $CD4^+$ T-cells	56-75	-
TE $CD4^+$ T-cells	-	20-35; 43-49; 68-72
$CD4^+$ Th1-cells	55-60	-

CD4⁺ Th2-cells	-	46-53
CD4⁺ Th17-cells	57-72	-
CD4/CD8 ratio	66-93	46-56
$\gamma\delta$–T-cells	40-49	82-93
MAIT/NKT	37-52	-
Plasmablast	67-93	-

*Numbers indicate age in year.

Abbreviations: DC: Dendritic cells, NK: Natural killer cells, EM: Effector memory, TM: Terminal effector, CM: Central memory, MAIT/NKT: mucosa-associated invariant T-cells/ Natural Killer T-cells.

Table S4: Age ranges outside of the 95% confidence band of the first derivative of the smoothing spline regressions (i.e., significant increase represents the age range located above zero of the 95% confidence band or decrease in immune cell counts represents the age range located below zero of the 95% confidence band).

**Immune Cell type**	**Female**		**Male**		**Negative age × sex interaction in females in comparison to males**
	Decrease	Increase	Decrease	Increase	
Neutrophil Granulocytes	-	20-60*	-	-	-
Non-classic Monocyte	-	20-61	-	20-57	-
Total dendritic cells	-	-	71-93	-	-
pDCs	60-60; 75-93	-	-	-	-
cDCS	-	-	65-93	-	-
Early NK cells	-	20-66	-	-	-
Late NK cells	-	20-72	-	59-93	-
Total $CD3^+$ T-cells	47-72	-	-	-	-
Total $CD8^+$ T-cells	20-53	-	-	-	20-33
Naive $CD8^+$ T-cells	20-93	-	20-93	-	20-62
$CD8^+$ T_{cm}-cells	-	-	-	20-46	20-45
$CD8^+$ T_{em}-cells	-	20-93	-	20-93	-
$CD8^+$ TEMRA	54-93	-	59-93	-	-
Total $CD4^+$ T-cells	54-93		59-93		
Naive $CD4^+$ T-cells	52-93	-	55-93	-	-
$CD4^+$ T_{em}-cells	43-93	-	48-93	-	-
$CD4^+$ TEMRA	-	20-63	-	20-72	-

CD4$^+$ Th2-cells	-	20-49	-	50-54	-
CD4$^+$ Th17-cells	58-93	-	59-93	-	-
CD4/CD8 ratio	-	20-52	65-93	-	-
Gamma delta T-cells	33-41	-	-	-	-
MAIT/NKT	20-93	-	-	-	50-93
Total B-cells	48-93	-	-	-	-
Naive B-cells	64-93	-	52-93	-	-
B_{mem} cells	20-80	-	-	-	20-56
Plasmablast	45-93	-	59-93	-	-

*Numbers indicate age in year. *Abbreviations: pDC: plasmacytoid dendritic cells, cDCs: conventional dendritic cells, NK: Natural killer cells, T_{em}: Effector memory, TEMRA: terminally differentiated effector memory RA+, T_{cm}: Central memory, MAIT/NKT: mucosa-associated invariant T-cells/ Natural Killer T-cells, B_{mem} cells: Memory B-cells.

Table S5: The smoothing spline regressions for identified clusters in deep immunophenotyping. Age ranges outside of the 95% confidence band of the first derivative of the (i.e., significant increase represents the age range located above zero of the 95% confidence band or decrease in immune cell percentage represents the age range located below zero of the 95% confidence band).

**Immune Cell type**	**Female**		**Male**		**Age × sex interaction in females in comparison to males**
	Decrease	Increase	Decrease	Increase	
B_{mem} cells (Cluster 2)	-	-	-	-	-
follicular B-cells (Cluster 11)	27-93	-	-	-	20-93
Naive B-cells (Cluster 13)	-	-	20-53	-	20-56
naïve CD4+ T-cell (Cluster 1)	51-93	-	54-93	-	-
cytotoxic effector CD8+ (Cluster 5)	-	20-93	-	53-93	-
activated CD4+ Teff cells (Cluster 9)	-	20-65	65-93	-	43-93
CD8+ TEMRA (Cluster 12)	-	20-93	-	20-93	-
CD8+ Teff cell (Cluster 18)	-	20-93	-	20-93	-
CD4+ Tcm cells (Cluster 20)	52-70; 78-93	-	20-70	-	-
MAIT/NKT (Cluster 24)	20-93	-	20-82	-	-
naïve CD8+ T-cells (Cluster 29)	20-93	-	20-93	-	-

*Numbers indicate age in year. *Abbreviations: T_{em}: Effector memory, TEMRA: terminally differentiated effector memory RA+, MAIT/NKT: mucosa-associated invariant T-cells/ Natural Killer T-cells, B_{mem} cells: Memory B-cells.

Table S6: Phenotypic characterization of major leukocyte lymphocytic lineage used for deep-phenotypic analysis

**Phenotypic assignment**	**Marker expression**
CD4^{+} naive	CD45RO^{-}, CD45RA^{+}, CCR7^{+}, CD38^{+}, CD27^{+}, CD127^{+}
CD4^{+} active effector	CD45RO^{-}, CD45RA^{+}, CCR7^{+}, CD38^{+}, CD27^{+}, CD127^{+}, CXCR3^{+}
CD4^{+} central memory	CD45RO^{+}, CD45RA^{-}, CCR7^{+}, CD28^{+}, CD38^{low}, CXCR5-, CCR6^{+}, CCR4^{+}, CD25^{-}, CD27^{+}, CD127^{+}, CXCR3^{+}
CD8^{+} cytotoxic effector	CD45RO^{-}, CD45RA^{+}, CCR7^{-}, CD56^{high}, CD161^{-}, CD57^{+}, CD28^{-}, CCR6^{+}, CD27^{-}, CD127^{-}, CXCR3^{+}
CD8^{+} terminal effector	CD45RO^{-}, CD45RA^{+}, CCR7^{-}, CD56^{-}, CD161^{-}, CD57^{+}, CD28^{-}, CCR6^{+}, CD27^{-}, CD127^{-}, CXCR3^{low}
CD8^{+} effector	CD45RO^{-}, CD45RA^{+}, CCR7^{-}, CD28^{-}, CD27^{low}, CD127^{low}, HLADR^{low}, CXCR3^{+}
CD8^{+} naive	CD45RO^{-}, CD45RA^{+}, CCR7^{+}, CD28^{+}, CD38^{low}, CD27^{+}, CD127^{+}, CXCR3^{+}
MAIT/NKT	CD45RO^{+}, CD45RA^{low}, CCR7^{-}, CD56^{+}, CD161^{high}, CD28^{+}, CCR6^{+}, CD27^{+}, CD127^{+}, CXCR3^{+}
follicular B-cell	CD27^{+}CD38^{low}IgD^{-}CD45RA^{+}CXCR3^{+}CXCR5^{+}CCR6^{+}HLADR^{+}CCR7^{low}
memory B-cell	CD27^{+}CD38^{high}IgD^{-}CD45RA^{+}CXCR3^{+}CXCR5^{+}CCR6^{+}HLADR^{+}CCR7^{high}
Naïve B-cell	CD27^{-}IgD^{+}CD45RA^{+}CXCR3^{-}CXCR5^{+}CCR6^{+}HLADR^{+}CCR7^{+}CD38^{+}

Abbreviations: MAIT/NKT: Mucosal associated invariant T-cells/Natural killer T-cells.
